# Point-of-Care Laboratory of Pathogen Diagnosis in Rural Senegal

**DOI:** 10.1371/journal.pntd.0001999

**Published:** 2013-01-17

**Authors:** Cheikh Sokhna, Oleg Mediannikov, Florence Fenollar, Hubert Bassene, Georges Diatta, Adama Tall, Jean-François Trape, Michel Drancourt, Didier Raoult

**Affiliations:** 1 URMITE, IRD 198, UM63, CNRS 7278, Inserm 1095, Aix Marseille Université, Marseille, France; 2 Campus commun UCAD-IRD d'Hann, Dakar, Senegal; 3 Institut Pasteur de Dakar, Dakar, Senegal; University of California Davis, United States of America

## Abstract

**Background:**

In tropical Africa, where the spectrum of the bacterial pathogens that cause fevers is poorly understood and molecular-based diagnostic laboratories are rare, the time lag between test results and patient care is a critical point for treatment of disease.

**Methodology/Principal Findings:**

We implemented POC laboratory in rural Senegal to resolve the time lag between test results and patient care. During the first year of the study (February 2011 to January 2012), 440 blood specimens from febrile patients were collected in Dielmo and Ndiop villages. All samples were screened for malaria, dengue fever, *Borrelia* spp., *Coxiella burnetii, Tropheryma whipplei, Rickettsia conorii, R. africae, R. felis*, and *Bartonella* spp.

**Conclusions/Significance:**

We identified DNA from at least one pathogenic bacterium in 80/440 (18.2%) of the samples from febrile patients. *B. crocidurae* was identified in 35 cases (9.5%), and *R. felis* DNA was found in 30 cases (6.8%). The DNA of *Bartonella* spp. was identified in 23/440 cases (4.3%), and DNA of *C. burnetii* was identified in 2 cases (0.5%). *T. whipplei* (0.2%) was diagnosed in one patient. No DNA of *R. africae* or *R. conorii* was identified. Among the 7 patients co-infected by two different bacteria, we found *R. felis* and *B. crocidurae* in 4 cases, *B. crocidurae* and *Bartonella* spp. in 2 cases, and *B. crocidurae* and *C. burnetii* in 1 case. Malaria was diagnosed in 54 cases. In total, at least one pathogen (bacterium or protozoa) was identified in 127/440 (28.9%) of studied samples. Here, the authors report the proof of concept of POC in rural tropical Africa. Discovering that 18.2% of acute infections can be successfully treated with doxycycline should change the treatment strategy for acute fevers in West Africa.

## Author Summary

In tropical Africa, clinical laboratories capable of performing complicated diagnostic studies like PCR are rare and are almost always found in large cities. Moreover, a number of infectious diseases, many of them are emerging and neglected, may be quickly and reliably diagnosed only by molecular biology. This is one of the reasons why the repertoire of bacterial infectious diseases in tropical Africa is poorly known. The laboratory based on the Point-of-Care (POC) principle has been designed in order to resolve the time lag between test results and patient care, which is the critical point for the treatment. We report here the first successful experience of the installation of POC laboratory in rural Senegal. During the first year of the study (February 2011 to January 2012) we identified DNA from at least one pathogenic bacterium in 80/440 (18.2%) of the samples from febrile patients. In most of the cases it was relapsing fever and rickettsiosis agents. Malaria was diagnosed in 54 cases. In total, at least one pathogen (bacterium or protozoa) was identified in 127/440 (28.9%) of studied samples. Discovering that at least 18.2% of acute infections can be successfully treated with doxycycline should change the treatment strategy for acute fevers in West Africa.

## Introduction

Each year in sub-Saharan Africa, more then 11 million people die [1], and, for the majority of individuals, the causes of death are largely uninvestigated. These uninvestigated deaths are generally attributed to infectious diseases. Access to reliable diagnostic testing is severely limited in sub-Saharan Africa, and misdiagnosis commonly occurs, although the diagnosis is essential to the prevention and treatment of disease [2]. Moreover, a number of infectious diseases, many of them are emerging and neglected, may be quickly and reliably diagnosed only by molecular biology.

Dielmo and Ndiop are two Senegalese rural villages where a longitudinal study of malaria for long-term investigations of host-parasite relationships and the mechanisms of protective immunity has been conducted since 1990 and 1993, respectively [3], [4]. The small and stable populations of Dielmo and Ndiop are closely followed by a research partnership between Dakar Pasteur Institute, Institut de Recherche pour le Développement (IRD) and the Senegalese Ministry of Health and Preventive Medicine. Malaria morbidity in Dielmo and Ndiop has been changing over the last 20 years [5]. Availability of rapid diagnostic tests (RDT), combination therapy and widespread use of insecticide-treated nets were the leading factors contributing to reduced malaria morbidity, which is similar to the reasons for the decrease in malaria mortality in other malaria-endemic African countries [5]–[7]. In Niger in 2009–2012, for example, the incidence of malaria among febrile patients seeking aid in the health facilities during the dry season was as low as 4% [8]. Though the incidence of malaria has declined, the diagnosis of the febrile illness in Africa still remains a challenge.

In 2008, we started to investigate the causes of non-malarial fevers in Senegal to identify appropriate strategies of case management in rural communities with declining rates of malaria. Our team was able to identify the different bacterial pathogens responsible for acute febrile syndromes in the Senegalese villages of Dielmo and Ndiop. The most intriguing feature of our studies was the identification of *Rickettsia felis* as the agent of the common febrile disease in rural Senegal [9], [10]. Similar results were obtained by an independent team of researchers in Kenya [11], [12]. The incidence of reports of this febrile illness in both countries is approximately 2–4 %, but in the rainy season in Senegal, it may actually be much higher (unpublished data). Tick-borne *Rickettsiae* species (*R. conorii, R. africae, R. massiliae, R. aeschlimannii, R. sibirica mongolitimonae*) were also identified in vectors and patients [13], [14]. The tick-borne relapsing fever caused by *Borrelia crocidurae* plays a very important role in the febrile morbidity in Senegal [15], and our findings confirmed the importance of this disease [16]. *Tropheryma whipplei*, the agent of Whipple's disease, may be responsible for some cases of febrile illness and diarrhea [17], [18]. We also found that the etiological agent of Q fever, *Coxiella burnetii*, is extremely frequent in both ticks and patients in rural Senegal, including in the villages of Dielmo and Ndiop [19]. Finally, we identified the trench fever agent *Bartonella quintana* in head lice in Senegal [20].

Based on this repertoire of bacterial species, we adapted the approach to fevers for the future, in order to include a wide systematic molecular biological exploration of patients with fever in Dielmo and Ndiop.

### Limits of the previously conducted studies and the basis of POC implementation in rural Senegal

The POC immuno-chromatographic tests (ICT) tests for malaria and HIV diagnostics are extensively used in Africa [21]. The malaria RDT played a significant role in reducing of malaria morbidity [5]. Other diagnostic tests are either not available, or, as in case of trachoma, not suitable for field diagnosis as the specificity decreases in hot and dry conditions [22].

Although POC tests rely mostly on ICT or agglutination assays, miniaturization and full automation of molecular methods allow for quicker real-time PCR-based detection of pathogens using simplified procedures. It was reported to be successfully applied in the POC diagnostics [23]. Although very promising experiences using molecular express diagnostic tests based on GenXpert technology were reported in South Africa for the diagnosis of tuberculosis [24], the availability of these tests in sub-Saharan Africa is still extremely insufficient. For this reason, all of the samples were initially collected in the rural health dispensaries and then sent to Marseille (France) for molecular studies. Sending the samples to France, however, limited or perhaps negated the direct diagnostic benefit of our studies. The time-consuming logistics and laboratory procedures hampered the use of available data for clinical needs.

The present paper provides the report of the installation of the POC laboratory based on ICT and real-time PCR-based detection of pathogens in rural Africa, the activity report and describes the methodology used to organize the running of this new biomedical technology in response to the needs of rural African healthcare.

## Materials and Methods

### Study sites

**Figure 1:**
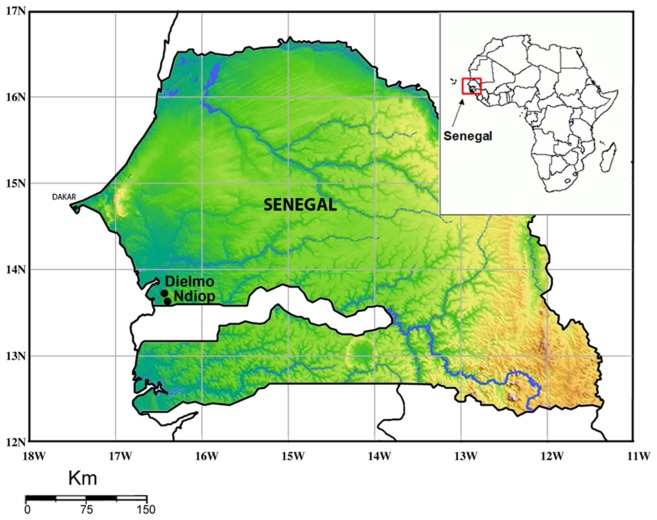
Location of Dielmo and Ndiop villages in Senegal, West Africa.

Located in the Saloum region of Senegal 280 km southeast of Dakar and approximately 15 km north of the Gambian border, Dielmo is an approximately 350-inhabitant village where malaria has been holoendemic. Ndiop with its ∼400 inhabitants is situated 5 km away (Figure 1; Table 1). The study site has been described in detail elsewhere [3]. Malaria transmission was intense and perennial, with a mean 258 infected bites per person per year during 1990–2006 [5].

**Table 1:** Summary data of the POC laboratory in Dielmo, Senegal.

Geographic coordinates	Dielmo: 13°43′N;16°24′W Ndiop: 13°41′N;16°22′W
Administrative situation	Senegal; Fatick region; Toubacouta rural community
Date of beginning of full-time activity	February 2011
Number of tests used	16 types of freeze-dried mix for real-time PCR; 3 RDT (malaria, dengue fever, influenza)
Number of samples studied	440
Number of positive tests	80
Total cost of project	158 670 euro

### The Dielmo field research station

**Figure 2:**
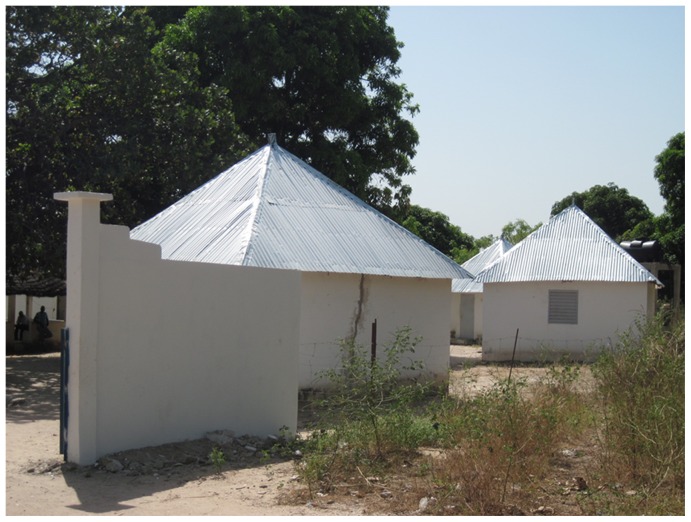
Health and clinical research station in Dielmo. A malaria longitudinal prospective study for the long-term investigation of host–parasite associations has been conducted in Dielmo since 1990 and the POC was established in 2011.

The research station is situated within 10 m of the Nema River and 30 m of the first household in the village and includes a dispensary with a laboratory and also has ten huts to accommodate the project staff and visitors (Figure 2). One nurse, two technicians and three fieldworkers are present every day in the village. The dispensary is open 24 hours per day, 7 days per week. The same type dispensary exists also in Ndiop. The dispensaries and the laboratory are equipped with standard materials for the screening and diagnosis of different conditions, including malaria and other febrile diseases (light microscopy, thick-smear, rapid malaria diagnostic test, hemoglobin diagnostics, stethoscope, blood pressure measurement, pregnancy testing) and a pharmacy that has essential medicines. The logistic equipment includes a generator, solar energy, one freezer at −20°C and one refrigerator at +4°C and one vehicle that is used for research operations and patient referrals. The station has a mobile telephone but does not have a reliable Internet connection.

### The concept of the POC test in rural Senegal

Point-of-Care (POC) is defined as medical diagnosis at or near the site of the patient [23]. We synthesized several principal issues concerning the installation of molecular-based POC in Dielmo:

1. For many important infectious diseases (spotted fevers, Q fever, relapsing fever etc.) the RDT are not available and the real-time PCR stays the most sensitive and available diagnostic method.
2. The analysis should be performed with several blood drops obtained from the finger tip. This procedure, contrary to the vein puncture, rarely encounters the refusal from the population.
3. All reagents, especially ready-to-use mixes, for the real-time PCR should be resistant to unstable transport conditions. Lyophilized reagents are the best choices.
4. The room for the POC should be specifically prepared and well constructed. It should be thermo-isolated, air conditioned and protected from insects and dust.
5. Electricity should be provided. In the absence of electricity, a diesel electric generator should be provided. Solar power is not usually sufficient for the functioning of the POC based on molecular methods.
6. The personnel should be specifically trained. The rotation of personnel between the study center and the POC should be guaranteed.

### Realization of the POC

In September 2010, we started to build the POC laboratory suite at the Dielmo field research station based on the proposed model [23]. In keeping with the design of PCR suites, the dimensions of the room of 9 m^2^ with a double brick-wall were designed to better isolate the room from the outside temperature. Air conditioning was assured by a split tropicalized air conditioner.

In November 2010, we started the installation of the equipment. The POC was equipped with working tables and chairs and with a portable meteorological station. The laboratory equipment included one freezer at −20°C and one refrigerator at +4°C, a safety cabinet designed for PCR rooms, a heating block for Eppendorf tubes, a manual polyvinyl chloride pump, a portable centrifuge for 1.5 ml tubes, a vortex, a Qiagen BioRobot EZ1 Workstation for DNA extraction, and two Smart cycler II units (Cepheid Europe; Maurens-Scopont, France) supplied with computers, printers and mini-centrifuges for specially designed tubes [23]. The pre-existed solar power was not usually enough to supply the energy for the molecular-based laboratory; the capacities of batteries do not allow to maintain the work of the real-time PCR machine, computer and air conditioner at the same time for at least one and half hours. A diesel power generator was installed outside to provide the energy for the POC. The total cost of the POC was estimated at 158,670 euros (all taxes and transportation included).

#### Staff

During the installation of the POC laboratory, we recruited and trained two laboratory technicians to be able to entirely manage the POC laboratory, including performing the direct diagnostic activities, managing the stocks of reagents and materials, managing the logistics of the samples and materials, and assuring the hygiene, security and environmental safety of the POC laboratory. The rotation of the personnel every three weeks is assured by the laboratory transport that is based in Dakar.

#### Logistics

The car permanently situated in the field assures the transport of the samples from Ndiop to Dielmo. The results of the tests are communicated by cell phone. The DNA samples are transported at 4°C in Dakar once every three weeks, during the rotation of the POC technicians.

#### Quality, hygiene, security and environmental protection

The control of the quality of diagnostic procedures is performed continuously. First, all of the DNA samples extracted from patients are stored in the refrigerator at +4°C. The samples are firstly transported at +4°C in Dakar once every three weeks by the IRD shuttle vehicle in the portable refrigerator. Then they are regularly transported to Marseille under the strict cold chain conditions, where the same diagnostic procedures are repeated. The control of the DNA extraction is provided by the qPCR of the human housekeeping gene, coding for β-actin [19]. All supplied lyophilized reagents are tested at the POC upon arrival with a cascade dilution of positive controls.

Entry into the POC is restricted to only the two laboratory technicians and the staff of the URMITE laboratory. To prevent the entry of the dust and insects, the POC laboratory is separated from the dispensary by a small atrium. Entry to the POC laboratory is permitted only if one is wearing surgical overshoes. All hygienic measures, including cleaning, are performed by technicians. The waste products are separated into combustible non-infectious waste (paper, cotton, etc.) that is burned in the station and potentially infected and sharps that are stored in special containers and sent back to Dakar for appropriate treatment.

#### Patients, samples and diagnostic techniques

**Figure 3:**
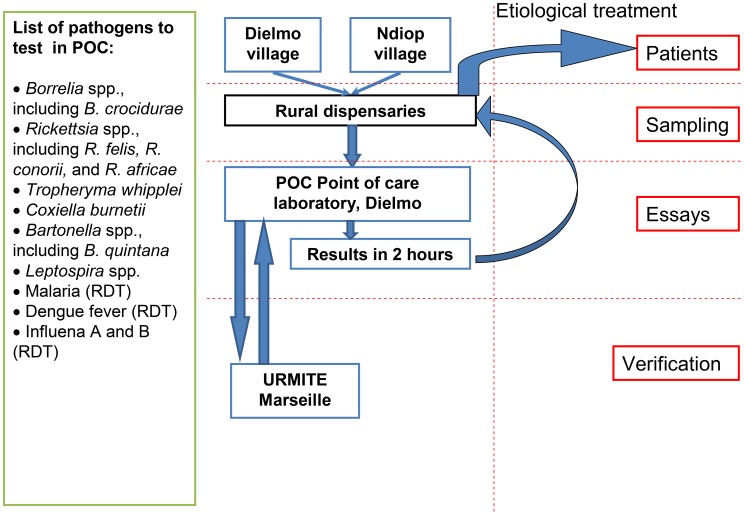
General scheme of the POC with a list of the pathogens diagnosed.

Patients eligible for inclusion in this study were all patients with fever (axillary temperature >37.5°C) who had visited the dispensaries in Dielmo and Ndiop, and for whom medical examinations were performed. A questionnaire was completed for each individual. In the dispensary, the Giemsa-stained thick blood smears are checked for plasmodia and *Borrelia* spp. At the same time, 3–4 blood drops (approximately 300 µl) are collected in a 1.5 ml tube that contains 20 µl of 0.2 M trisodium citrate. This sample is used for immuno-chromatographic tests and DNA extraction. Malaria tests were performed with Core Malaria rapid tests (common antigens for all Plasmodium species, *P. vivax*- and *P. falciparum*-specific antigens) (Core Diagnostics, Birmingham, UK). Tests for Dengue fever (ICT for antigens and antibodies) from serum are performed with Dengue Duo tests (SD, Korea). The details of patients included and the organization of the POC are shown in Figure 3.

The DNA from the patients' blood was extracted using the Biorobot EZ1 Workstation (Qiagen, Courtaboeuf, France), with a customized extraction protocol following the manufacturer's instructions. The DNA samples were stored at 4°C until they were used in the PCR amplifications. Bacterial DNA was detected by quantitative PCR reactions that were performed using Smartcycler equipment and software (Cepheid Europe; Maurens-Scopont, France).

The total time lag between the samples registration and the results for all tests (ICT and real-time PCR) is evaluated in 2 hours maximum.

#### Preparation of lyophilized reagents

Ready-to-use for qPCR freeze-dried mixes were prepared in the laboratory of molecular biology in URMITE, Marseille, at the request of the POC technicians and were transferred on a monthly basis to Senegal [25]. We added trehalose at final concentration of 0.2 M/l [26] to stabilize and enhance the qPCR reaction. Each tube with lyophilized mix was prepared with enough volume for 4 reactions (undiluted and 1/10 diluted patient sample, positive and negative controls).

**Figure 4:**
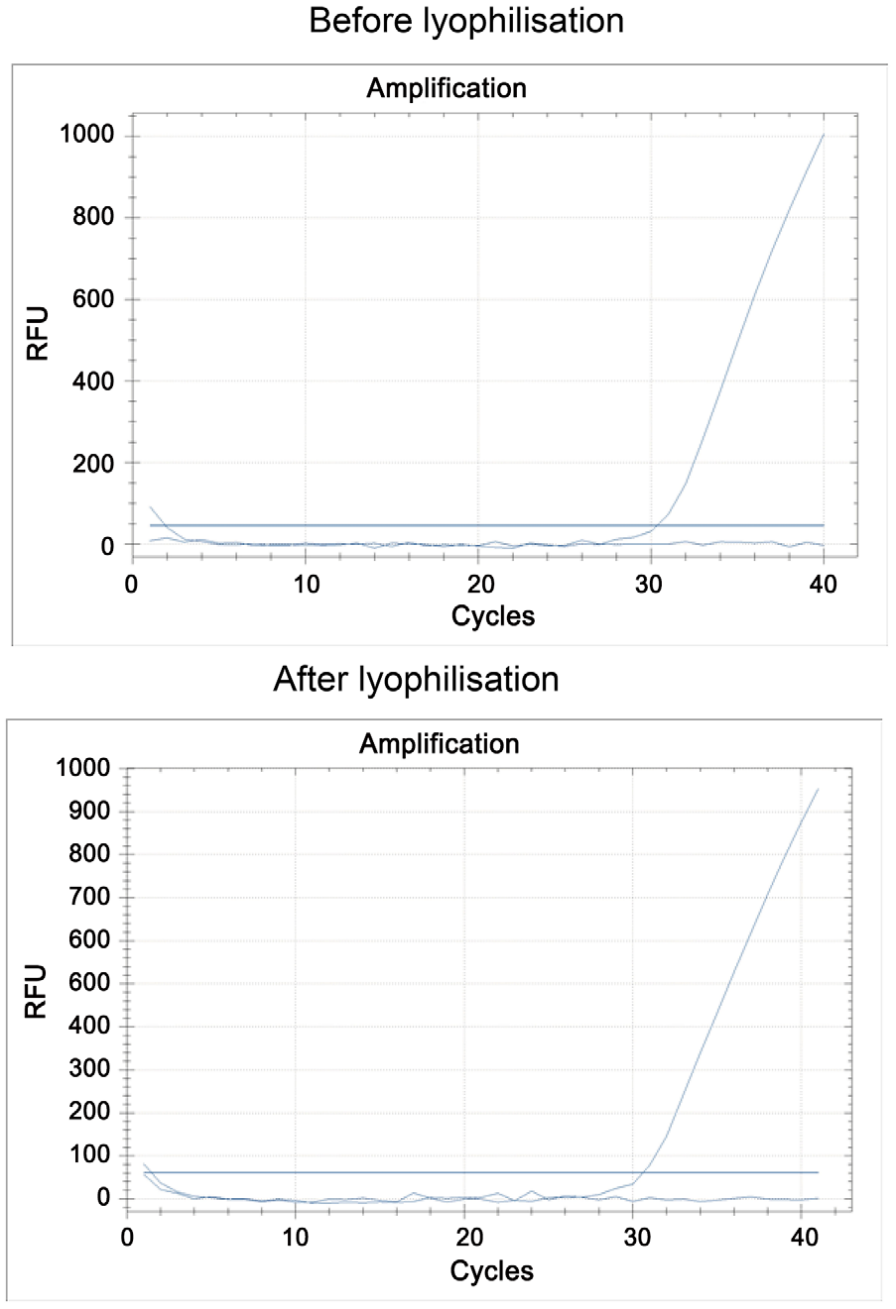
Real-time PCR curves of freshly prepared and lyophilized mixes for the detection of *R. felis*.

The quality of the lyophilization was regularly tested. No difference in sensitivity was noted when the mix was tested before and after lyophilization (Figure 4). The lyophilized mixes can be stored for at least two months at 20°C without a decrease in their activity (data not shown).

The repertoire of the freeze-dried mixes is presented in Table 2. We used *Rickettsia* genus-specific qPCR [9], a *Rickettsia felis*-specific biotin synthase gene-based system [9], a *Borrelia* genus-specific 16S rRNA gene-based system [16], a *Coxiella burnetii*-specific system based on intergenic spacers [19] and a *Tropheryma whipplei*-specific system based on repeated sequences [27] as well as *Rickettsia typhi*-, *R. africae*- and *R. conorii*-specific qPCR (Table 2). All primers sets were previously tested in Marseille for specificity and sensitivity. The samples were considered positive if the Ct (cycle threshold) number did not exceed 35 that corresponded to the ability to reveal 10–20 copies of bacterial DNA [19].

**Table 2:** Primers and probes used in the study.

Forward (F) and reverse (R) primers and probes (P) for screening samples					Forward (F) and reverse (R) primers and probes (P) for confirmation of positive samples				
Organism	Gene		Primers (5′-3′) and probes (5′-6-FAM – TAMRA -3′)	Ref	Organism	Gene		Primers (5′-3′) and probes (5′-6-FAM – TAMRA -3′)	Ref
*Rickettsia* spp. spotted fever group	*gltA* (RKND03)	FRP	GTGAATGAAAGATTACACTATTTAT GTATCTTAGCAATCATTCTAATAGC CTATTATGCTTGCGGCTGTCGGTTC	[14]	*Rickettsia* spp. spotted fever group	RC0338 (1029)	FRP	GAMAAATGAATTATATACGCCGCAAA ATTATTKCCAAATATTCGTCCTGTAC CGGCAGGTAAGKATGCTACTCAAGATAA	[14]
					*Rickettsia conorii*	Hypothetical protein gene RC0743	FRP	TTGGTAGGCAAGTAGCTAAGCAAA GGAAGTATATGGGAATGCTTTGAA GCGGTTATTCCTGAAAATAAGCCGGCA	This study
					*Rickettsia africae*	spoT15-dam2 intergenic spacer	FRP	TGCAACACGAAGCACAAAAC CCTCTTGCGAAACTCTACTTTTGA CGTGTGGATTCGAGCACCGGA	This study
*Rickettsia felis*	*Biotin synthase*	FRP	ATGTTCGGGCTTCCGGTATG CCGATTCAGCAGGTTCTTCAA GCTGCGGCGGTATTTTAGGAATGGG	[9]	*Rickettsia felis*	*OrfB*	FRP	CCCTTTTCGTAACGCTTTGCT GGGCTAAACCAGGGAAACCT TGTTCCGGTTTTAACGGCAGATACCCA	[9]
*Rickettsia typhi*	Hypothetical protein gene 01310	FRP	TGTCAGATTATAAAGACGATGCTCAGA GCAGCTTGTACTCCTTTAATTTGTTC CCGCTACCGCAAATCCATCAGA	This study					
*Borrelia* spp.	16S rDNA	FRP	AGCCTTTAAAGCTTCGCTTGTAG GCCTCCCGTAGGAGTCTGG CCGGCCTGAGAGGGTGAACGG	[16]	*Borrelia* spp.*	*flagelin*	FR	GCTGAAGAGCTTGGAATGCAACC TGATCAGTTATCATTCTAATAGCA	[34]
*Coxiella burnetii*	Spacer IS1111	FRP	CAAGAAACGTATCGCTGTGGC CACAGAGCCACCGTATGAATC CCGAGTTCGAAACAATGAGGGCTG	[19]	*Coxiella burnetii*	Spacer IS30a	FRP	CGCTGACCTACAGAAATATGTCC GGGGTAAGTAAATAATACCTTCTGG CATGAAGCGATTTATCAATACGTGTATGC	[19]
*Bartonella* spp.	16S-23S internal transcribed spacer ITS2)	FRP	GGGGCCGTAGCTCAGCTG TGAATATATCTTCTCTTCACAATTTC CGATCCCGTCCGGCTCCACCA	[35]	*Bartonella* spp.	16S-23S internal transcribed spacer (ITS3)	FRP	GATGCCGGGGAAGGTTTTC GCCTGGGAGGACTTGAACCT GCGCGCGCTTGATAAGCGTG	[35]
					*Bartonella quintana*	yopP	FRP	TAAACCTCGGGGGAAGCAGA TTTCGTCCTCAACCCCATCA CGTTGCCGACAAGACGTCCTTGC	[35]
*Tropheryma whipplei*	WiSP family protein (WHI3)	FRP	TTGTGTATTTGGTATTAGATGAAACAG CCCTACAATATGAAACAGCCTTTG GGGATAGAGCAGGAGGTGTCTGTCTGG	[18]	*Tropheryma whipplei*	WiSP family protein (WHI2)	FRP	TGAGGATGTATCTGTGTATGGGACA TCCTGTTACAAGCAGTACAAAACAAA GAGAGATGGGGTGCAGGACAGGG	[18]

* - PCR coupled by sequencing, performed in Marseille only.

#### Ethic statement

The Ministry of Health and Preventive Medicine of Senegal and the assembled village population initially approved the project protocol in 1990. Since granting ethical approval in 2000, 2005 and 2009, the National Ethics Committee of Senegal has conducted three site visits, and ad-hoc research committees of the Ministry of Health and Preventive Medicine, the Dakar Pasteur Institute and the IRD provided recommendations to continue the project. Ethical approval is renewed on a yearly basis. Following introduction of POC diagnosis, written informed consent was obtained from all of the adult residents in the study villages and from the guardians of children under 15 years of age after careful explanation of the goals of the project to the assembled village population. The National Ethics Committee of Senegal approved the most recent protocol, including the POC for three years (May 2010–May 2013).

**Figure 5:**
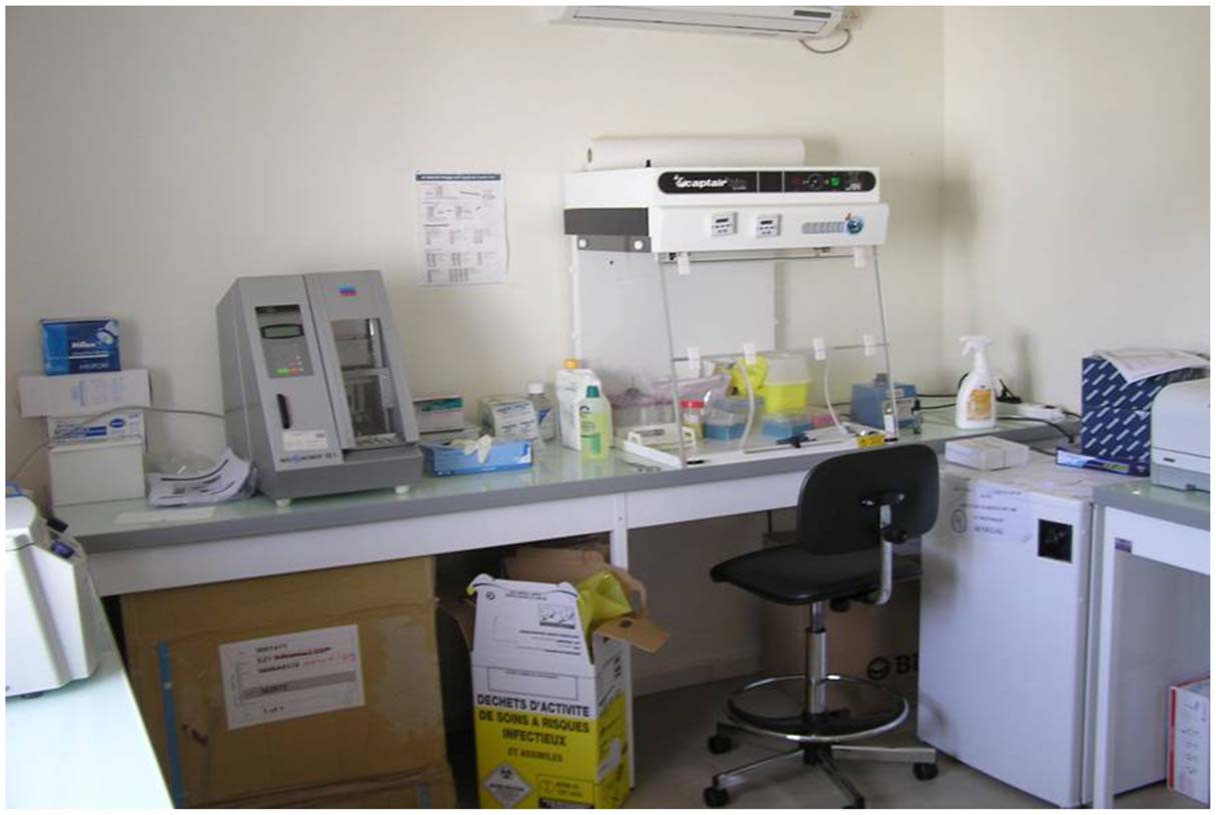
Point-of-Care laboratory implemented in Dielmo village, showing the DNA extraction and mix preparation materials.

The POC was officially inaugurated in the presence of the local authorities and the Director of our unit at the end of November 2010. Due to several organizational difficulties and the need for some constructional improvements, the range of diagnostic procedures became completely available in February 2011 (Figure 5). The running costs of the POC in the conditions of pre-existing field research station and IRD implantation in Dakar (including the local salaries of technicians, transport, energy, and preparation of the lyophilized reagents) may be roughly estimated in 25,000 euros per year.

## Results

During the first year of the study (February 2011 to January 2012), 440 blood specimens from febrile patients were collected in Dielmo and Ndiop villages. All samples were screened for malaria (rapid diagnostic test), dengue fever (rapid diagnostic test), *Borrelia* spp., *C. burnetii*, *T. whipplei, R. conorii, R. africae, R. felis*, and *Bartonella* spp. A second round of qPCR was performed to confirm positive results of the first reaction for *C. burnetii*, *T. whipplei* and *Bartonella* spp.

We identified at least one pathogenic bacterium DNA in 80/440 (18.2%) of the samples from febrile patients. No mortality was associated with this group of patients during our study. Infection with *B. crocidurae* was identified in 35 cases (9.5%). Different results were found at the POC in Marseille in four cases. In two of these, the borderline Ct number (34.38 and 34.29) was identified in Dielmo, and the samples were considered positive, but the samples were counted as negative in Marseille because the qPCR presented a Ct value higher than 35. In the other two cases, the samples were negative in Dielmo but weakly (37.88 and 37.77) positive in Marseille (Table 3). However the sequences of amplicons corresponded to *B. crocidurae*, and the samples were considered positive.

**Table 3:** Comparison between the molecular assays performed in Marseille and in Dielmo.

	Number of positive / Number of tested			Sensitivity	Specificity	Positive predictive value	Negative predictive value
	Marseille	Dielmo	Discrepancies between both	of on-field POC compared to assays performed in Marseille as gold standard			
*Rickettsia felis*	28/440	30/440	2^1^	100%	99.5%	93.5%	100%
*Rickettsia conorii*	0/440	0/440	0	NA	100%	NA	100%
*Rickettsia africae*	0/440	0/440	0	NA	100%	NA	100%
*Coxiella burnetii*	2/440	2/440	0	100%	100%	100%	100%
*Tropheryma whipplei*	1/440	1/440	0	100%	100%	100%	100%
*Bartonella* spp., including *B. quintana*	23/440	23/440	0	100%	100%	100%	100%
*Bartonella quintana*	4/440	Not done	NA	NA	NA	NA	NA
*Borrelia crocidurae*	35/440	35/440	4^2^	94.3%	99.5%	94.3%	99.5%

The sensitivity, specificity, positive predictive value, and negative predictive value of on-field Point-Of-Care (POC) were compared to molecular assays performed in Marseille as gold standard.

1 Two patients concluded as positive in Dielmo were concluded as negative in Marseille.

2 Two patients concluded as positive in Dielmo were concluded as negative in Marseille and two patients concluded as negative in Dielmo were concluded as positive in Marseille.

NA: Not available.

*R. felis* DNA was found in 30 cases (6.8%) of acute febrile illness in Dielmo and Ndiop. All samples that generated a fluorescent signal for *R. felis* in qPCRs performed in Marseille were previously *R. felis* positive in Dielmo. However, in 11/30 cases the Ct numbers were higher than 36. We found discordance in only two cases between a positive result in POC and a negative result in Marseille. Interestingly that in 3 cases observed by one of the authors (O.M.), the infection presented with a rash in the contrary to our previous data [9]. No cases of *R. typhi* infection was diagnosed.

The DNA of *Bartonella* spp. (including *B. quintana*) was identified in 23/440 cases (5.2%). Unfortunately, the *B. quintana*-specific tests were not available at the beginning of this study, so we were only able to identify the species after the specific qPCR was performed in Marseille. In four of 23 cases, the *Bartonella* species was identified as *B. quintana*. There were no discordance between Marseille and Senegal results

The DNA of *C. burnetii* was identified in 2 cases (0.5%). Both were confirmed in Marseille.

*T. whipplei* (0.2%) was diagnosed in one patient. No DNA of *R. africae* or *R. conorii* was found in patients.

We calculated sensitivity, specificity, positive and negative predictive values for each of used tests (Table 3). Due to the low number of positive samples, the specificity and sensitivity in most cases were 100%.

Among the 7 patients co-infected with two different bacteria, *R. felis* and *B. crocidurae* were present in 4 cases, *B. crocidurae* and *Bartonella* spp. were found 2 cases, and *B. crocidurae* and *C burnetii* were found in 1 case. In 54 cases we identified *Plasmodium falciparum* in the samples. In 5 cases DNA of *R. felis* was also found in the sample, in one case *B. crocidurae* and in one case *T. whipplei*. No samples were found positive for dengue fever. In total, at least one pathogen (bacterium or protozoa) was identified in 127/440 (28.9%) of studied samples.

The appropriate treatment for the etiological agent of disease was applied when possible [5]. All of the identified bacteria are susceptible *in vitro* or *in vivo* to doxycycline [9], [14], [16], [28]. The dose for the children was calculated based on the amount of 5 mg/kg per day (Table 4). No pregnant women were diagnosed with bacterial infections.

**Table 4:** The treatment of patients included in the study (440 samples).

Disease	Number of cases	Treatment
**Malaria**	54	Combination therapy
***R. felis*** ** infection**	30	5 days of 200 mg per day of doxycycline
**Relapsing fever**	35	5 days of 200 mg per day of doxycycline
**Bartonellosis.**	19	5 days of 200 mg per day of doxycycline
**Q fever**	2	15 days of 200 mg per day of doxycycline
***T. whipplei*** ** infection**	1	15 days of 200 mg per day of doxycycline

As all included patient participate in the long-term malaria surveillance program [3] we were able to monthly survey almost all patients but without retesting them for bacterial pathogens if they had no fever. Fourteen persons left the protocol due to death or migration: 1 person died of hepatic cirrhosis and 13 have left the villages. In all cases the treatment (Table 3) resulted in rapid resolution of all symptoms, including the fever. No relapses and mortality associated with identified infectious diseases was registered.

## Discussion

### Infectious diseases diagnostics and POC implantation

Molecular diagnosis of infectious diseases supplemented and even replaced many current culture and serologic tests in microbiology [29]. Many fastidious bacteria (including rickettsiae, *C. burnetii*, bartonellae, *T. whipplei*) are very difficult to isolate. For many infectious diseases (including borrelioses and *T. whipplei* infection) serology is not reliable method for diagnostic [30]. Moreover, for all these infection serological methods of acute infection are difficult and require highly experienced technician. Thus, molecular methods play the primary role in diagnostics of these infectious diseases in traditional and POC-based laboratories [23]. The widespread use of qPCR that is less expensive than conventional PCR and reduces delay in the diagnosis of rickettsial infections. The development of qPCR strategies in the diagnosis of rickettsioses has previously been proposed [31]. Thus, qPCR-based on-site molecular testing POC laboratory seems to be the easiest way to diagnose infectious diseases in rural conditions.

### Freeze-drying of real-time PCR mixes

Molecular diagnostics are a very sensitive method of diagnosing infectious diseases. Materials and reagents are fragile, and the contamination of the reagents by amplicons may be a significant problem [30], [31]. Lyophilized, ready-to-use mixes for individual tests might be the solution for the POC in rural Africa. Each flask contains the freeze-dried mix for the testing of one sample (with dilution; positive and negative controls), is thermo-stable, easy-to-transport and is protected against contamination. During the entire study, we never had a problem with contamination. Our negative controls were consistently negative.

### Healthcare issues

The impact of POC for the health of the population may be important. Up until now, the clinical diagnosis of non-malarial, acute febrile illnesses in rural Senegal was impossible. The example of tick-borne relapsing fever is interesting because before the implementation of the POC laboratory in Dielmo, *Borreliae* were detected in Giemsa-stained thick blood smears during the examination for malaria. However, only very experienced laboratory personnel could confirm the presence of *Borreliae* in the blood; thus, in most cases, the diagnosis was made only after repeated examination performed in the URMITE Dakar laboratory. This means that there was a delay of several weeks between the original consultation and the diagnosis. A feature of relapsing fever is that it may last for weeks without appropriate treatment. Therefore, the patient might still need medical assistance even 2–3 weeks after the initial consultation.

As for the other pathologies, before the beginning of our project, they were either unknown in Senegal (infections caused by *R. felis, R. africae*, *T. whipplei*, and *B. quintana*) or were rarely studied and never diagnosed (Q fever). The current practice in Senegal is to use amoxicillin and/or cotrimoxazole in case of non-malarial fevers [32]. Discovering that all identified infections can be successfully treated with doxycycline and many of them are not sensitive to amoxicillin and/or cotrimoxazole should change the treatment strategy for acute unexplained fevers in West Africa.

### Perspectives and limitations of the study

POC laboratory based on ICT and real-time PCR is a viable in locations with very limited infrastructure. However, the installation of this type of laboratory by a public health system may be limited by the finance and expertise. The costs of installation and running may be quite high for the developing countries. The running demands the qualified technicians, regular supervision and a good QC system that is not always available.

The POC laboratory, however, may be used as a very effective tool for studying the epidemiology of infectious diseases. The diagnosis of virtually any infectious disease may be performed in a very short period of time. This rapid diagnosis may be very important for the global healthcare systems. The solutions proposed by POC may be the only tool for discovering the etiologies in cases of emerging and novel infectious diseases and for studying the epidemiology of poorly known or poorly studied infections.

The example of infection caused by *R. felis* in Africa is very interesting. The emergence of this bacterium as a frequent cause of febrile disease in Senegal and Kenya [9], [11], [12] and the simultaneous absence of the bacterium in fleas [33], which are considered the normal vectors of the bacterium worldwide, suggest that in Africa, the epidemiology of the febrile illness caused by *R. felis* may be different from that in other countries. We already possess some evidence that the clinical picture of primary infection may have some unique traits, such as a vesicular rash (data not published). The POC laboratory may help to discover the enigmas of this infection in Africa. For example, it can help in the discovery of the vector and reservoirs of the bacterium and in defining the clinical picture of the illness.

Moreover, POC laboratories may be easily arranged by broad-range diagnostic systems to perform large spectrum studies for potentially unknown pathogens. These laboratories may be very important for global healthcare and travel medicine.

Finally, we can conclude that the establishment of the POC laboratory in rural Senegal may indicate the beginning of a new approach in the studies of emerging tropical infectious diseases. The close contact between the patient and the researcher may provide diagnosis of disease in a very short time. Additionally, the plasticity of the assays makes it possible to quickly change the specialization of the POC and to broaden the specificity of the molecular studies performed at the POC.
