# Correction: Point-of-care laboratory of pathogen diagnosis in Rural Senegal

**DOI:** 10.1371/journal.pntd.0014233

**Published:** 2026-04-22

**Authors:** The PLOS Neglected Tropical Diseases Editors

This Correction resolves the prior Expression of Concern [2] for this article [1].

Following the publication of the article and Expression of Concern [1,2], PLOS investigated the concerns pertaining to the reported ethical oversight and the article’s adherence to PLOS research ethics policies.

Specifically, the Materials and Methods section of this article [1] reports that blood samples were collected from febrile patients who had visited dispensaries in Dielmo and Ndiop, Senegal, between February 2011-January 2012. The ethics statement of the article reports that the project protocol was initially approved by the Ministry of Health and Preventative Medicine of Senegal in 1990, and that the approval was renewed on an annual basis, but the article does not report ethics approval reference numbers.

A representative of the Aix-Marseille Université Ethics Committee stated that the institutional investigation into the ethics concerns concluded this article meets ethical standards. They stated that the area where these samples were collected is subject to a long-term longitudinal study that started in 1990, and that a dispensary was set up with the aim of studying malaria whilst also providing care to the local population. The representative stated that during the longitudinal study various research teams intervened in these villages, in agreement with the local authorities, to carry out follow-up studies. They provided the documents N° 00.86 MSP/DS/CNERS and N° 001380 MSP/DS/DER for editorial review.

- Document N° 00.86 MSP/DS/CNERS is an ethics approval document issued on June 2, 2010, by the Ministère de la Santé et de la Prévention of the République de Sénégal for protocol SEN21/09 involving a study titled *“Identification des agents pathogènes responsables de fièvre au Sénégal. Réalisation de tests diagnostiques chez les malades consultant dans les dispensaires de Dielmo et Ndiop”.* It grants a one-year approval for carrying out diagnostic tests on patients in the Dielmo and Ndiop dispensaries with the aim of identification of pathogens responsible for fever in Senegal.
- Document N° 001380 MSP/DS/DER is an administrative authorization issued on May 31, 2011, by the Ministère de la Santé et de la Prévention of the République de Sénégal for a study titled *“Identification des agents pathogènes responsables de fièvre au Sénégal. Réalisation de tests diagnostiques chez les malades consultant dans les dispensaires de Dielmo, Ndiop, Niakhar, Mlomp, Bandafassi et Keur Momar Sarr”* and grants administrative authorization for one year to implement the studies described in protocols SEN21/09 and SEN37/09.

In light of the information provided by the institute, the final sentence of the first paragraph of the Materials and Methods subsection Ethic statement. is updated to:

The protocol for this study was approved by the Senegalese Ministry of Health and Preventive Medicine on June 2, 2010, and May 31, 2011 (N° 00.86 MSP/DS/CNERS and N° 001380 MSP/DS/DER).

PLOS notes that Document N° 00.86 MSP/DS/CNERS requires the removal of doxycycline as systematic treatment for cases of fever. Therefore, all statements related to the doxycycline treatment findings and treatment strategies reported in the abstract’s Conclusions/Significance subsection, the Results section, and the Discussion’s Healthcare issues subsection of this article [1] should be interpreted with caution.

With this update, the *PLOS Neglected Tropical Diseases* Editors consider the ethics approval concerns resolved. This Correction supersedes the prior Expression of Concern [2].
